# *GBA* mutation promotes early mitochondrial dysfunction in 3D neurosphere models

**DOI:** 10.18632/aging.102460

**Published:** 2019-11-21

**Authors:** Constanza Morén, Diana Luz Juárez-Flores, Kai-Yin Chau, Matthew Gegg, Glòria Garrabou, Ingrid González-Casacuberta, Mariona Guitart-Mampel, Eduardo Tolosa, María José Martí, Francesc Cardellach, Anthony Henry Vernon Schapira

**Affiliations:** 1 Cellex, IDIBAPS, University of Barcelona-Hospital Clínic of Barcelona, Barcelona 08036, Spain; 2 Centro de Investigación Biomédica en Red (CIBER) de Enfermedades Raras (CIBERER), Madrid 28029, Spain; 3 Department of Clinical and Movement Neurosciences, UCL Queen Square Institute of Neurology, University College London, London NW3 2PF, UK; 4 Neurology Department, Hospital Clínic of Barcelona, Barcelona 08036, Spain

**Keywords:** Gaucher's disease, Parkinson's disease, neurospheres, mitochondria, autophagy

## Abstract

Glucocerebrosidase (*GBA*) mutations are the most important genetic risk factor for the development of Parkinson disease (PD). *GBA* encodes the lysosomal enzyme glucocerebrosidase (GCase). Loss-of-GCase activity in cellular models has implicated lysosomal and mitochondrial dysfunction in PD disease pathogenesis, although the exact mechanisms remain unclear. We hypothesize that *GBA* mutations impair mitochondria quality control in a neurosphere model.

We have characterized mitochondrial content, mitochondrial function and macroautophagy flux in 3D-neurosphere-model derived from neural crest stem cells containing heterozygous and homozygous *_N370S_GBA* mutations, under carbonyl cyanide-m-chlorophenyl-hydrazine (CCCP)- induced mitophagy.

Our findings on mitochondrial markers and ATP levels indicate that mitochondrial accumulation occurs in mutant *_N370S_GBA* neurospheres under basal conditions, and clearance of depolarised mitochondria is impaired following CCCP-treatment. A significant increase in TFEB-mRNA levels, the master regulator of lysosomal and autophagy genes, may explain an unchanged macroautophagy flux in *_N370S_GBA* neurospheres. PGC1α-mRNA levels were also significantly increased following CCCP-treatment in heterozygote, but not homozygote neurospheres, and might contribute to the increased mitochondrial content seen in cells with this genotype, probably as a compensatory mechanism that is absent in homozygous lines.

Mitochondrial impairment occurs early in the development of GCase-deficient neurons. Furthermore, impaired turnover of depolarised mitochondria is associated with early mitochondrial dysfunction.

In summary, the presence of *GBA* mutation may be associated with higher levels of mitochondrial content in homozygous lines and lower clearance of damaged mitochondria in our neurosphere model.

## INTRODUCTION

Homozygous mutations in the *GBA* gene encoding the lysosomal enzyme glucocerebrosidase (GCase) cause Gaucher disease (GD), the most common lysosomal storage disorder (LSD). *GBA* mutations are also numerically the most important known genetic risk factor for Parkinson disease (PD) [6, 39]. The most frequent *GBA* mutation associated with PD is N370S (*_N370S_GBA*) [6, 39]. Mutations in *GBA* decrease GCase activity, leading to defects in autophagic-lysosomal function and a-synuclein aggregate accumulation.

Cell models of dermal fibroblasts or transformed lymphocytes (lymphoblasts) have been used to understand the cell biology associated with neurodegenerative diseases, including PD [10, 19, 41] but the utility of non-neural cells to provide insight into the cellular basis of neurodegeneration is limited [22]. The development of stem cell-derived models of disease, such as embryonic stem cells [13, 20] and induced pluripotent stem cells (iPSCs) from patients with PD are considered more appropriate in this respect [33]. Collections of neural stem cells, also known as neurospheres, have been developed from patients with neurodegenerative diseases, including PD [21, 28, 31]. Neurospheres have been used as a tool to model neurodegenerative disorders [21], developmental studies [1, 32], cell differentiation [47] and regenerative medicine [23]. Neural stem cell derived neurospheres have an advantage over embryonic/iPSCs, as they do not require genetic reprogramming. Neurospheres can be obtained by using pluripotent neural crest stem cells (NcSC) from adipose tissue [33, 43] and have been previously used in PD research [14].

Mitochondrial dysfunction has been associated with aging and several neurodegenerative diseases including PD [35, 36], and several lines of investigation are directed towards increasing mitochondrial biogenesis and respiration as neuroprotective strategies [3]. Mitochondrial autophagy (mitophagy) has been shown to contribute to the pathogenesis of genetic forms of PD [15]. Mutations in genes that regulate mitophagy, such as PTEN-induced-putative-protein-kinase 1 (*PINK1*) and *PARK2*/*Parkin*-mutations cause early onset PD [25]. Following a loss of mitochondrial membrane potential (MMP) (↓ΔΨm), PINK1 accumulation in the outer membrane acts as a sensor for mitochondrial damage and induces translocation of cytosolic Parkin, through a mechanism that requires the kinase activity of PINK1 [25]. Parkin ubiquitinates several mitochondrial proteins which aids the recruitment of damaged mitochondria to phagophores by binding to LC3-II directly, or via the adaptor protein p62 [45]. It is not clear whether p62 is required for Parkin mediated mitophagy [8, 24]. An increasing body of evidence implicates defects in quality control pathways in both GD and PD, but the precise role of mitochondrial function and mitophagy in the pathogenesis of these diseases remains to be elucidated [5, 7, 26, 37]. Transcription factor EB (TFEB) regulates autophagy by activating the genes that encode lysosomal hydrolases, lysosomal v-ATPase pumps, lysosomal regulators and autophagy regulators and so it is considered a master regulator of autophagy that has emerged as a potential therapeutic target for PD. TFEB has also been associated with activation of peroxisome proliferator-activated receptor gamma coactivator 1-alpha (PGC1α), a regulator of mitochondrial biogenesis. It has been reported that cells with increased TFEB protein have significantly higher PGC1α mRNA levels, resulting in increased mitochondrial content. These findings suggest that TFEB is activated following mitophagy to maintain the autophagy-lysosome pathway and mitochondrial biogenesis [11].

The characterization of mitochondrial function and autophagy in neurospheres would help to clarify whether a cause-effect relationship between the presence of *GBA* mutations and mitochondrial dysfunction occurs before neuronal differentiation. We hypothesise that *GBA* mutations likely impair the clearance of damaged mitochondria, promoting their accumulation, as a result of abnormal autophagy flux processing or biogenesis.

Mitophagy pathways could be altered in *_N370S_GBA* carriers due to defective recycling machinery and thereby changes in mitochondrial content. Therefore we have investigated the effect of *_N370S_GBA* mutations on macroautophagy and mitochondrial function in a 3D-neurosphere model derived from NcSC, in either basal conditions or following induction of mitophagy by the CCCP uncoupler.

## RESULTS

Homozygous, heterozygous and control donor subjects were age and sex matched, (Supplementary Table 1).

### Characterization of the neurosphere model

We first followed the morphological changes of NcSC upon induction to neurospheres. Cell aggregates began to form within 24 hours and the spherical structures grew in size as time progressed (48, 72 and 96 hrs). (Supplementary Figure 1).

**Figure 1:**
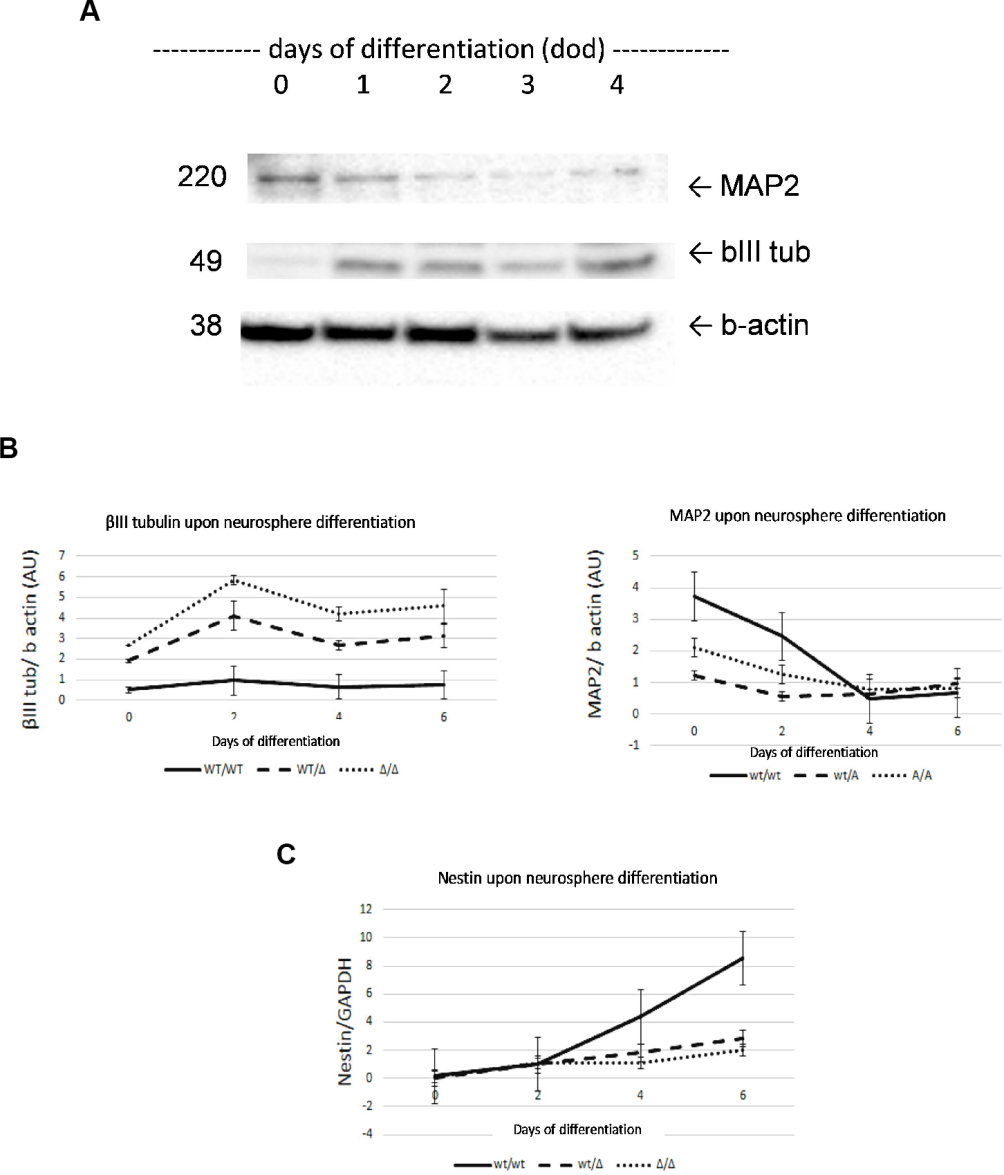
**Characterization of neurosphere development by analysis of β-III tubulin, MAP2 and nestin markers.** Neuronal markers β-III tubulin and MAP2 were determined by Western Blot (**A**). Both neural markers showed stabilized expression on day 4 of development. β-III tubulin and MAP2 levels confirmed neural properties of neurospheres (**B**). Increasing nestin mRNA levels confirmed neural stem cell properties of neurospheres during development (**C**). No significant differences were found at 4 day of development AU, arbitrary units. Results are expressed by mean± SEM.

**Figure 2:**
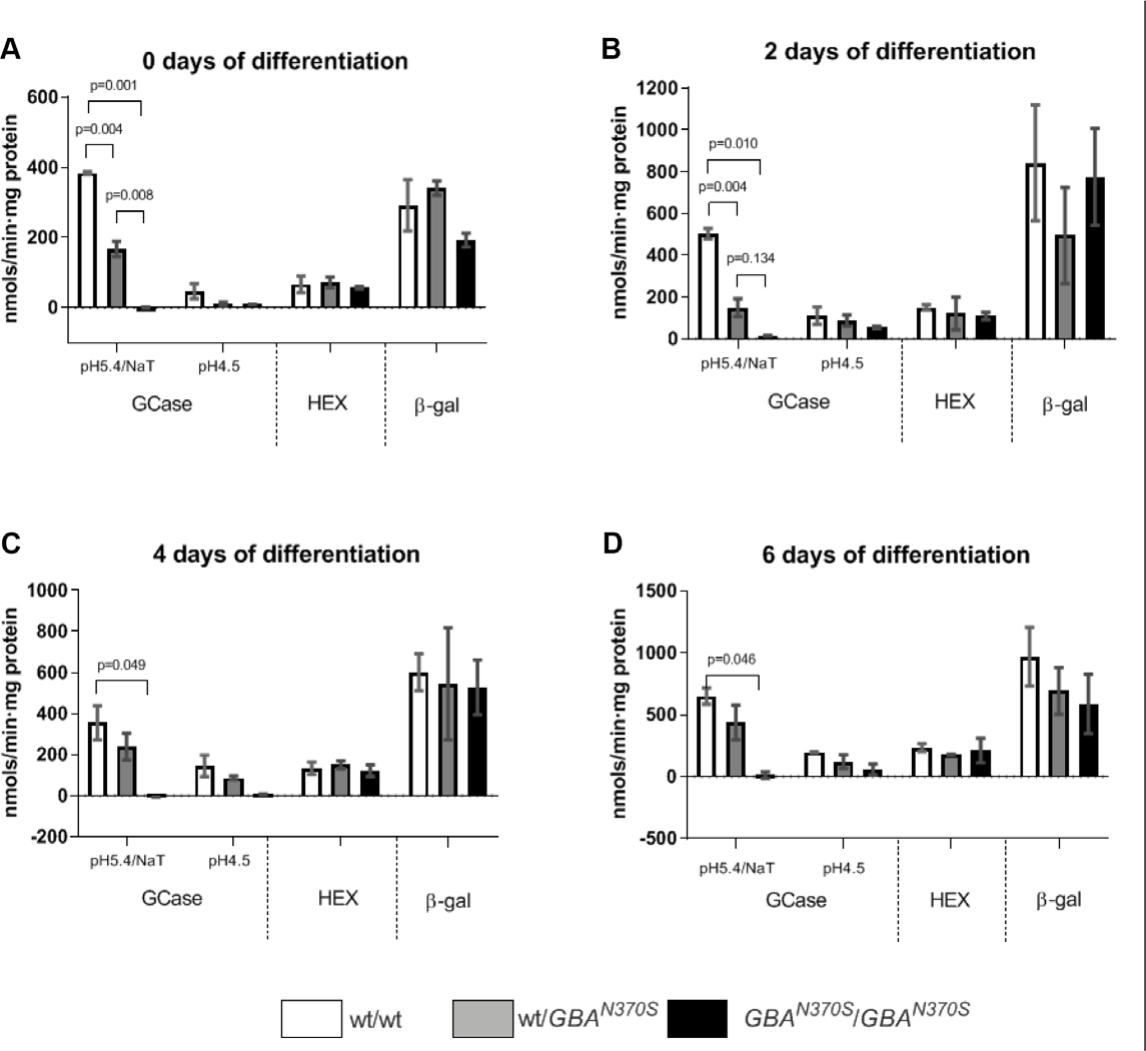
**GCase, HEX and β-gal enzymatic activities stabilized from 0 to 6 days of differentiation.** GCase activity decreased as expected for the respective genotypes (33% decrease in wt/*_N370S_GBA* and 98% decrease in *_N370S_GBA*/*_N370S_GBA*) when measured at pH 5.4 in the presence of sodium taurocholate. Measurement of GCase at pH 4.5 in the absence of the GCase activator sodium taurocholate yielded similar results, while HEX and β-gal were unaffected. Results are expressed by mean± SEM.

Next, we measured the protein level of neuronal markers i.e. β-III tubulin and Microtubule-associated protein 2 (MAP2) (Figure 1A and B), and the mRNA level of the stem marker nestin (Figure 1C), as well as GCase, HEX and β-gal enzymatic activities (Figure 2) of the 6 lines over time, to identify the length of time taken for the neurospheres to become biochemically stable, and to compare the influence of *_N370S_GBA* zygosity on neurosphere formation. We chose 4 days as the duration for the studies as MAP2 and β-III tubulin were stable at this time and nestin levels were not significantly different between the lines. Quantification of GBA protein levels are shown in Supplementary Figure 4. GCase activity showed a trend to reduction by 33% (p=NS) in wt/*_N370S_GBA* and was 98% decreased (p=0.049) in *_N370S_GBA*/*_N370S_GBA* when compared to controls, when measured at pH 5.4 in the presence of sodium taurocholate. As a confirmation, measurement of GCase at pH 4.5 in the absence of the GCase activator sodium taurocholate yielded similar results, while HEX and β-gal were unaffected.

### Validation of uncoupler treatment to dissipate ΔΨ and to induce mitophagy in the neurosphere model

**Figure 3:**
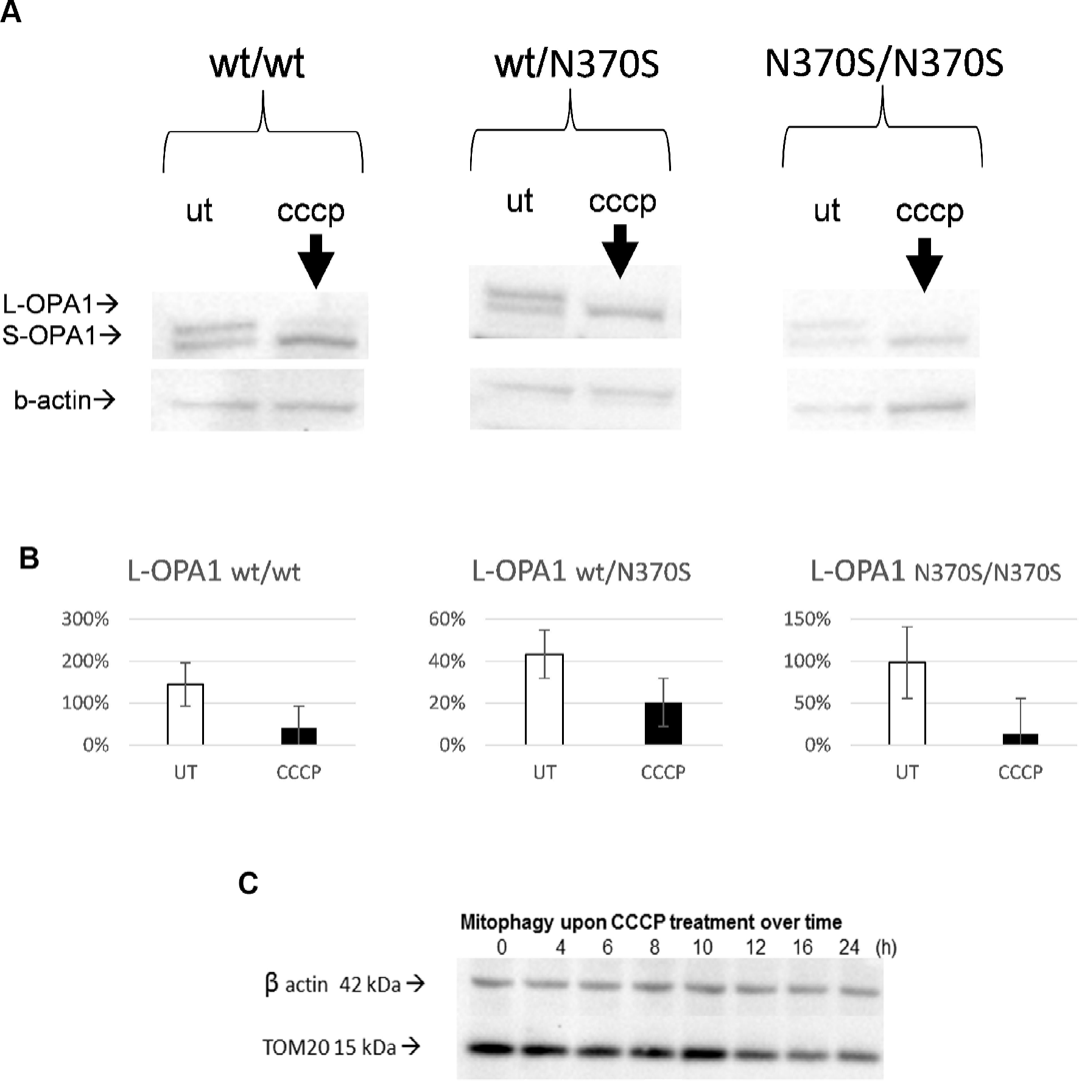
**Confirmation of mitochondrial uncoupling with CCCP in the neurosphere model.** Representative blots of OPA1 isoforms. Expected changes from long to short OPA1 isoforms were observed upon uncoupler treatment in all the genotypes, as mitochondria undergo depolarisation/fission following mitochondrial uncoupling (24h, 10 μM CCCP) (**A** and **B**). TOM20 levels progressively decrease from 0 to 24h in a control line (**C**). L-OPA1, long isoform of optic atrophy 1 protein OPA1; S-OPA1, short isoform of OPA1; UT, untreated.

Mitochondrial depolarisation following mitochondrial uncoupling was confirmed by loss of the long isoform of optic atrophy protein 1 (OPA1) (L-OPA1; Figure 3A and 3B) as previously described [9, 17, 30, 40] and was similar in all three genotypes. Mitochondrial content as measured by TOM20 protein level was decreased in a control cell line during the 24h CCCP treatment (Figure 3C); the progressive response over the time of treatment confirms the effect of CCCP-induced depolarization.

### Mitochondrial content following mitophagy induction in mutant *GBA* neurospheres

**Figure 4:**
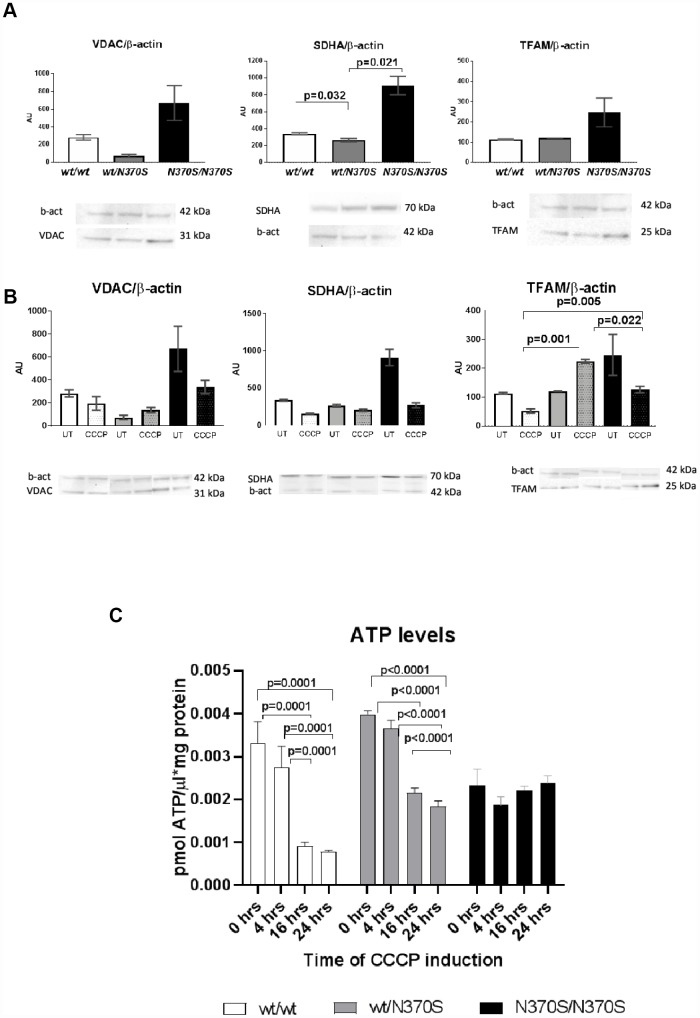
**Mitochondrial content under basal conditions in control, wt/ *_N370S_GBA* and *_N370S_GBA*/*_N370S_GBA* neurospheres was assessed by western blotting for the mitochondrial proteins VDAC1 (outer mitochondrial membrane), SDHA (inner mitochondrial membrane), and TFAM (matrix), normalized by β-actin content.** All markers tended to increase in *_N370S_GBA*/*_N370S_GBA* neurospheres in basal conditions, when compared to control lines (**A**) (SDHA was significant). As expected in control lines, protein levels of VDAC1, SDHA and TFAM and ATP levels decreased following CCCP treatment for 24 hours (**B** and **C**). Despite an apparent increase in mitochondrial mass in untreated conditions, ATP levels remained unchanged in wt/*_N370S_GBA* and *_N370S_GBA*/*_N370S_GBA* neurospheres (**C**). Mitochondrial markers of wt/*_N370S_GBA* did not show a marked response to CCCP uncoupling, in fact, TFAM significantly increased in the heterozygous lines and the decrease in ATP levels were not so pronounced (**B** and **C**) and ATP levels of homozygous *_N370S_GBA*/*_N370S_GBA* neurospheres showed a null response to CCCP uncoupling. All of the above data suggest that mitochondrial function may be impaired in *GBA* mutant neurospheres. AU: arbitrary units. Thin line indicates p value <0.05. Results are expressed by mean± SEM.

We compared the steady-state levels of mitochondrial content between different genotypes. As shown in Figure 4A, there was a significant increase in the levels of succinate dehydrogenase complex subunit A (SDHA), and a non-significant increase for voltage- dependent anion-selective channel 1 (VDAC) or mitochondrial transcription factor A (TFAM) in the homozygous lines.

As expected, trends to a reduction of protein level in a panel of mitochondrial markers representing the outer, inner membrane or mitochondrial matrix i.e VDAC1, SDHA and TFAM were present upon uncoupler treatment in the control lines (wt/wt) (Figure 4B) and ATP levels also decreased (Figure 4C). Despite the increase of mitochondrial content (SDHA subunit) under basal conditions, steady state levels of ATP in *_N370S_GBA/_N370S_GBA* presented trends to lower levels than control cells suggesting mitochondrial dysfunction (Figure 4C). After uncoupler treatment, ATP levels also decreased in the control lines. However, the decrease of mitochondrial markers were not so evident in *wt/_N370S_GBA* heterozygous lines and the response of ATP levels upon induced mitophagy was either partial or not observed in the *wt/_N370S_GBA* heterozygous and homozygous *_N370S_GBA* neurospheres, respectively. In fact, in the heterozygous line, the mitochondrial marker TFAM was significantly increased (Figure 4B).

**Figure 5:**
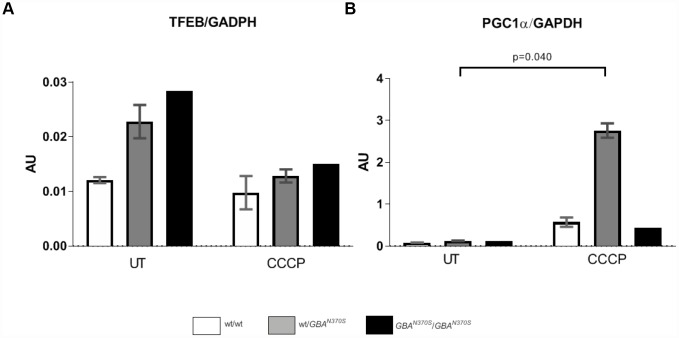
**Steady-state mRNA levels of TFEB and PGC1α during uncoupling treatment.** TFEB mRNA levels tended to decrease in wt/*_N370S_GBA*, and *_N370S_GBA*/*_N370S_GBA* but PGC1α significantly increased upon CCCP induction in wt/*_N370S_GBA*, probably in an attempt to compensate for the underlying mitochondrial defects. UT, untreated. AU: arbitrary units. Thin line indicates p value <0.05. Results are expressed by mean± SEM.

Since mitochondrial content is not only maintained by mitochondrial turnover but also mitochondrial biogenesis, we examined the latter by measuring mRNA levels of PGC1α transcript and one of its upstream regulators TFEB [11, 38] in neurospheres untreated and treated with CCCP for 24 hours. As displayed in Figure 5, mitochondrial depolarization led to a substantial and significant increase in PGC1α transcript in the heterozygous line compared to the control and homozygous lines. The steady-state level of TFEB mRNA expression was found inversely proportional to the GCase level, although not significantly (Figures 2 and 5). Treatment of wt/*_N370_GBA* or *_N370S_GBA*/*_N370S_GBA* neurospheres with CCCP resulted in similar TFEB mRNA levels, compared to control. PINK1 mRNA levels were similar in all the cell lines (data not shown) suggesting if there is impaired mitophagy it is probably not due to lower PINK1 levels (wt/wt 0.068±0.006, wt/*_N370S_GBA* 0.086±0.010, *_N370S_GBA*/*_N370S_GBA* 0.058±0.059).

### Macroautophagy in neurospheres carrying *_N370S_GBA* mutation

**Figure 6:**
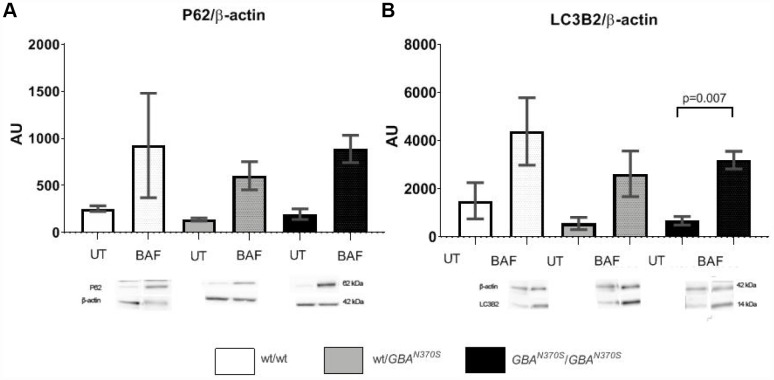
**Autophagy flux measurement.** Macroautophagy flux showed trends to further increase in the control lines. p62 and LC3-2 levels are similar in the 3 untreated cell lines, however, when autophagy was blocked under bafilomycin treatment, p62 and LC3-2 levels tended to increase the most in controls. The lack of significant differences suggests that preexistent mitochondrial impairment may account for the pathogenesis of *GBA* mutant neurospheres. AU, arbitrary units; UT, untreated; BAF, bafilomycin. Results are expressed by mean± SEM.

To investigate if mitophagy was impaired in neurospheres with GCase deficiency, we measured macroautophagy flux by LC3-II levels, a marker of autophagosome number, and sequestrosome/p62 (p62) levels (which helps bind both cargo and LC3-II in autophagosomes). Under basal conditions, p62 and LC3-II levels were similar in all groups (Figure 6). Following bafilomycin A1 (BAF) treatment, which prevents the fusion of autophagosomes to lysosomes, p62 and LC3-II increased in all groups as expected (Figure 6). However, there was no significant difference between the groups treated with BAF, suggesting that macroautophagy flux is not noticeably affected, which is in line with similar synuclein protein levels observed at day 4 of development (Supplementary Figure 3).

A summary of the raw data obtained from the experimental work is available in Supplementary Table 2.

## DISCUSSION

In this study neurospheres have been used to explore mitochondrial content, function and macroautophagy in the context of *GBA* mutations.

Mitochondrial abnormalities have been associated with *GBA* mutations [6, 12, 26, 42], and recent data have also shown that mitochondrial dysfunction is present in post-mortem tissues of PD patients, cell and animal models with the L444P *GBA* mutation [16]. Our study was designed to monitor steady-state levels of mitochondrial content, alterations in content upon mitochondrial depolarization induced mitophagy; the latter validated by loss of Ψm, decreased mitochondrial proteins, a decrease in total ATP and a shift of OPA1 isoforms in control neurospheres.

Our data indicate that heterozygous and homozygous *_N370S_GBA* produced different patterns of modified mitochondrial content. Homozygous *GBA* mutations tended to increase mitochondrial content under basal conditions (significantly increased SDHA levels were observed), while uncoupling resulted in decreased clearance of mitochondrial content. Intriguingly, heterozygous *GBA* mutations tended to increase mitochondrial content following mitochondrial uncoupling, and this was coincident with a large increase in PGC1α mRNA levels, a master regulator of mitochondrial biogenesis. This could be a compensatory mechanism for heterozygous lines, which homozygous lines under the same insult are not able to perform. Our macroautophagy flux data did not indicate a significant problem with the formation of autophagosomes with lysosomes in mutant neurospheres, suggesting that, if mitophagy is impaired in these cells, it could be due to excessive mitochondrial biogenesis, as observed by the PGC1α increase [2], rather than the autophagic machinery required for the degradation of damaged mitochondria. However, it is possible that we have not been able to detect macroautophagy defects due to the low sample size. Nevertheless, the fact that macroautophagy is not noticeably affected is consistent with the absence of differences in synuclein levels at day 4 of neurosphere development. The association of the loss of GCase activity and an increase in synuclein levels has been consistently observed and, in our model homozygous lines showed trends to higher levels of synuclein, while heterozygous lines showed intermediate levels over the controls, at several points of their development. Mitochondrial fission, as measured by the disappearance of the long isoform of OPA1, was similar between lines, suggesting additional alterations of mitochondrial turnover. PINK1 mRNA levels were also similar, and we were unable to detect either endogenous PINK1 or parkin protein levels (data not shown) suggesting either PINK1/parkin mitophagy occurs at an undetectable rate in our model, or does not occur at all in neurospheres, although we cannot discard technical reasons underlying the lack of the detection of these proteins. Mitochondria in *GBA* KO mice cortical neurons have been shown to recruit GFP-tagged parkin upon depolarization to a similar level as controls [27]. Other mitophagy initiators [4, 46] might be required for mitophagy in neurospheres. For instance, other proteins related to mitophagy such as BNIP3, FUND1, SMURF1 and BCL2-L-13 could also be affected but are beyond the scope of this study.

GCase deficiency in neuronal models is typically associated with an impairment of macroautophagy flux [5, 18, 37]. The increased mRNA levels of TFEB, a master regulator of lysosomal and autophagy genes [34] is the most likely explanation why we do not see changes in macroautophagy flux. Perhaps neurons are unable to maintain this compensatory mechanism, as they develop, particularly if mitochondrial dysfunction is occurring in these cells, and changes in autophagy flux are observed in more mature neurons.

**Figure 7:**
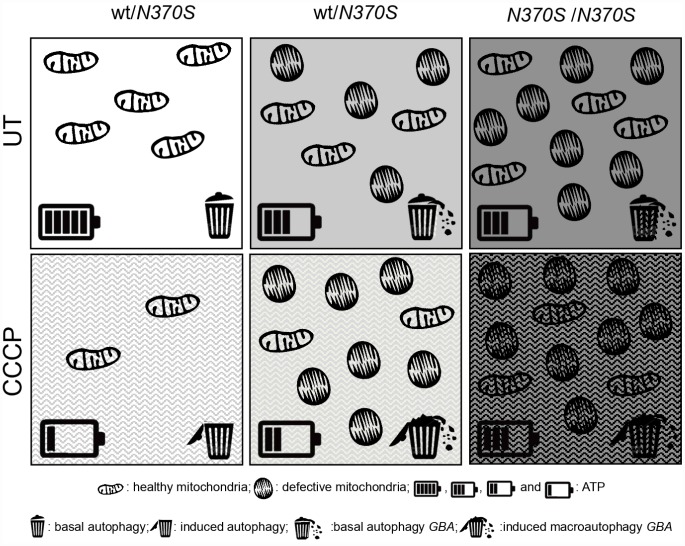
**Global summary of the observed molecular parameters in *GBA* mutant neurospheres.** Compared to the controls, dysfunctional mitochondrial accumulation occurs in mutant *GBA* neurospheres under basal conditions, as shown by the accumulation of healthy and altered mitochondria, represented as elongated and rounded structures, respectively. Mitochondrial elimination is correct under mitophagy induction (CCCP) in the control line, as observed by the decrease of ATP, represented by the battery, and the macroautophagy induction, represented by the garbage bin. There is an impaired turnover of depolarized mitochondria under induced mitophagy in *GBA* neurospheres. This is not a result of impaired autophagosome formation and degradation, but at an earlier stage, where defective mitochondrial dysfunction was observed.

In summary, as illustrated in Figure 7, dysfunctional mitochondrial accumulation occurs in mutant *GBA* neurospheres under basal conditions. Our data suggest that the impaired turnover of depolarized mitochondria is not a result of impaired autophagosome formation and degradation, but has its origin at an early stage, when defective mitochondrial function was observed.

## CONCLUSIONS

Neurospheres are a suitable model to study lysosomal storage disorders. We show that mitochondrial dysfunction is an early event preceding macroautophagy flux, at least in this model of neurospheres containing pathogenic *GBA* mutations.

Neurospheres might also be considered as a potential tool for drug discovery or cell replacement therapy because these cells can proliferate and differentiate into new neurons, astrocytes, and oligodendrocytes [14].

## MATERIALS AND METHODS

### Patients

All patients and controls included in the study signed an informed consent. The study has been approved by the institutional ethics committee Royal Free Research Ethics Committee (REC number 10/H0720/21). Methods have been performed in accordance with the appropriate guidelines and regulations of University College of London.

### Cell culture and treatment

Neural crest stem cells were obtained as previously described [44]. 6 cell lines corresponding to homozygous (*_N370S_GBA*/*_N370S_GBA*, n=2), heterozygous (wt/*_N370S_GBA* n=2) and control (wt/wt, n=2) subjects were grown in DMEM media supplemented with 10% fetal bovine serum, 1mM pyruvate, 0.5 ml uridine (50mg/ml) and 0.5% penicillin-streptomycin-fungizone. Then, cells were detached with accutase and cultured in DMEM-F12 (1:1) containing the following supplements and growth factors, 1x B27, 20μL leukemia inhibitor (10ng/mL), basic fibroblast growth factor (40ng/mL FGF2), epidermal growth factor (10ng/mL EGF) and 1% penicillin-streptomycin-fungizone.

Mitochondrial depolarization-mediated mitophagy was induced for 24 hours with 10 μM CCCP. Bafilomycin treatment was performed for 4 hours at 0.5 μM. Cells were washed twice with phosphate-buffered saline (PBS) and collected for analysis as described below.

### Western blot analysis and densitometry

Neurospheres were lysed and placed on ice for 15 min with RIPA buffer supplemented with 1x Halt protease inhibitor cocktail (Pierce). Cell lysates were centrifuged at 21,000g at 4°C for 5min and soluble material was retained for Western Blot analysis.

Protein levels were determined by using a bicinchoninic acid (BCA) kit (Pierce Thermo Fisher; Basingstoke, UK).

Equal amounts of 20 μg protein from the soluble material were resolved under reducing conditions in either: i) NuPAGE 4–12% polyacrylamide precast gels (Invitrogen, Carlsbad, CA, USA) using the 2 (N morpholino) ethane sulphonic acid (MES) buffer or: ii) NuPAGE 12% (Invitrogen, Carlsbad, CA, USA) using MOPS buffer and transferred onto Immobilon polyvinylidene difluoride membrane (PVDF) (Millipore, Watford, UK). Blocking was made with 10% skimmed milk powder in PBS. Antibodies detecting microtubule associated protein 2 (MAP2) (1:1000 MAP2, Invitrogen), βIII tubulin (1:1000 ab7751 Abcam), p62 (1:1666 ab56416 Abcam), β actin (1:10000, Sigma), TFAM (1:1000 Thermo Fischer Scientific), TOM20 (1:1000 Santa Cruz), VDAC1 (1:1000 ab14734 Abcam), SDHA (1:1000 ab14715 Abcam), OPA1 (1:500 BD/612606) and LC3 (1:1000 Cell Signalling) were used. Horseradish peroxidase conjugated secondary antibodies against mouse and rabbit IgG were used (1:2000) (Dako, Glostrup, Denmark). ECL reagent (GE Healthcare, Bucks, UK) was used to develop the blots and signals detected through the Chemidoc MP System (BioRad) and band densities measured using ImageLab analysis software. Data were normalized to β-actin levels.

### Lysosomal enzyme assays

GCase, beta-hexosaminidase (HEX) and beta-galactosidase (β**-**gal) enzymatic activities were determined at 37 °C. CE-sensitive GCase activity (end-point measurement) was determined as described [29] at pH 5.4 using 4-methylumbelliferyl-β-d-glucopyranoside as substrate in a plate reader and its activity is reported in the presence of the activator sodium taurocholate (Sigma). The increase in fluorescence of released 4-methylumbelliferone at 460 nm following excitation at 360 nm was followed after 1 h (Synergy, LabTech; Brighton, UK). Total HEX and β-gal were assayed with 4-methylumbelliferyl-2-acetoamido-2-deoxy-6-sulpho-b-D-glucopyransoside and 4-methylumbelliferyl-B-D-galactopyranoside as substrates, respectively. Enzymatic activities were normalised to the amount of proteins of the samples. Results were expressed as nmol/hour or minute/mg protein. A full length blot is shown in Supplementary Figure 2.

### ATP quantification

ATP Bioluminescence was measured in a microplate by the ATP Bioluminescence Assay Kit CLS II (Roche), following manufacturer’s instructions. Briefly, different dilutions of samples and serial diluted ATP standards were included as an internal curve in a black opaque microplate. Then a volume of 50μl luciferase reagent was added and luminescence was immediately measured in a synergy device (Synergy, LabTech, Brighton, UK). ATP levels were normalised to the amount of total proteins of the samples. Results were expressed as pmol/mg protein.

### Levels of mRNA

Following treatment, RNA was extracted from cells using RNeasy kit (Qiagen). RNA was converted to cDNA (Primer Design, Southampton, UK) and relative mRNA levels were measured using SYBERgreen (Applied Biosystems, Paisley, UK). Relative expression of Nestin (forward ACCAAGAGACATTCAGACTCC and reverse CCTCATCCTCATTTTCCACTCC), TFEB (forward CCAGAAGCGAGAGCTCACAGAT and reverse TGTGATTGTCTTTCTTCTGCCG, PGC1α (forward CAGAGAACAGAAACAGCAGCA and reverse TGGGGTCAGAGGAAGAGATAAA) and PINK1 (forward GGACGCTGTTCCTCGTTA and reverse ATCTGCGATCACCAGCCA). mRNA was measured with Power SYBRgreen kit (Applied Biosystems) using a STEP One PCR device (Applied Biosystems). GAPDH (forward GAAGGTGAAGGTCGGAGT and reverse GAAGATGGTGATGGGATTTC) mRNA levels were used to normalise data. Relative expression was calculated using the ΔCT method.

### Statistical analysis

Low sample size only allowed us to use replicates for comparisons. One way ANOVA followed by Bonferroni’s post-hoc correction was performed using SPSS Version 20 and GraphPad Version 8 to assess whether differences between groups were present. Results were expressed as mean ± SEM and significance was set at *p* value < 0.05.

### Ethics approval

All patients and controls included in the present study have presented their informed consent and the study has been approved by the institutional ethics committee Royal Free Research Ethics Committee (REC number 10/H0720/21). Methods have been performed in accordance with the appropriate guidelines and regulations of University College of London.

## Supplementary Material

Supplementary Figures

Supplementary Tables
