# Supplementary material for: GBA mutation promotes early mitochondrial dysfunction in 3D neurosphere models
Source: Aging (Albany NY). 2019 Nov 21;11(22):10338–55. doi: 10.18632/aging.102460 (PMC6914435; doi:10.18632/aging.102460)
Supplement: Supplementary Figures [file aging-11-102460-s002..pdf]

SUPPLEMENTARY FIGURES

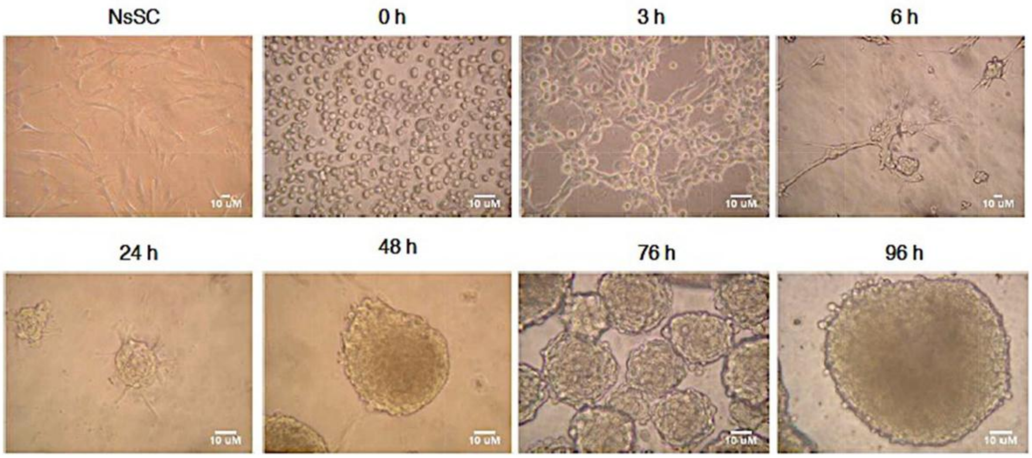

Supplementary Figure 1. Neurosphere formation and morphology from neural crest stem cells to 4 days of development. NcSC, neural crest stem cells.

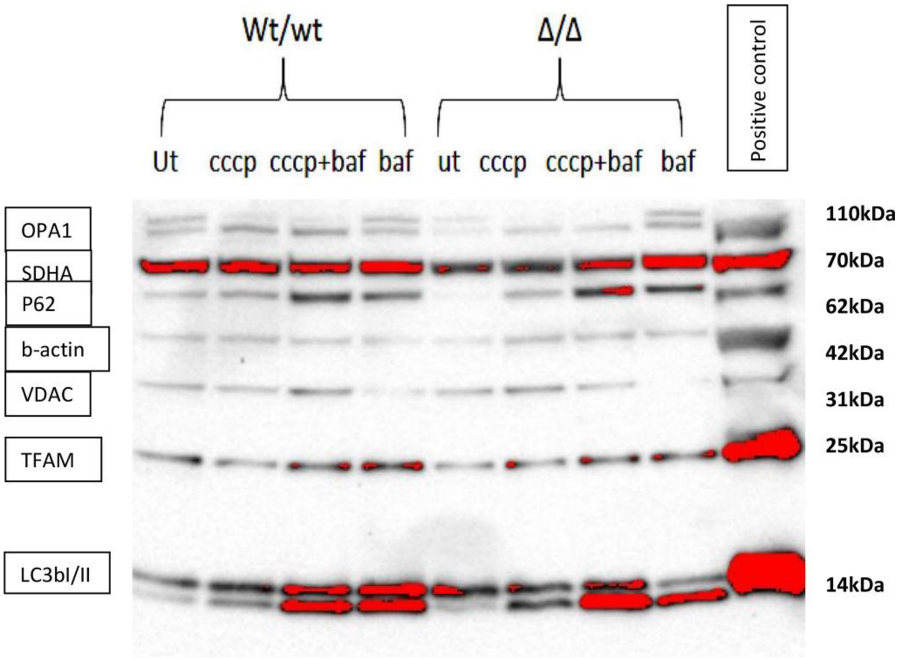

Supplementary Figure 2. Full-length blot corresponding to cropped data on OPA-1, SDHA, p62, b-actin, VDAC, TFAM and LC3BI/II, showing different specific tested antibodies in the same blot (from the top to the bottom of the blot): OPA-1, SDHA, P62, b-actin, VDAC, TFAM, LC3b-I/II…) represented in Figures 3, 4 and 6. Protein levels are quantified in each case before overexposure occurs.

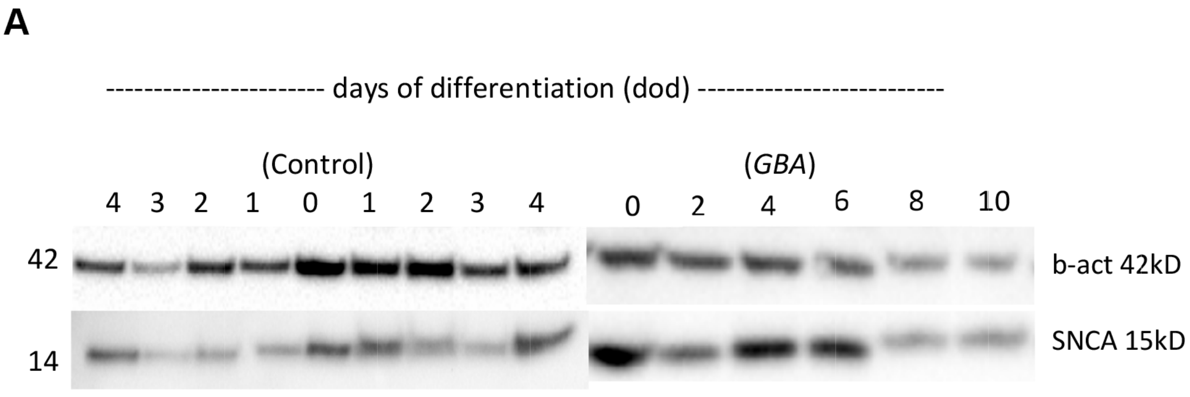

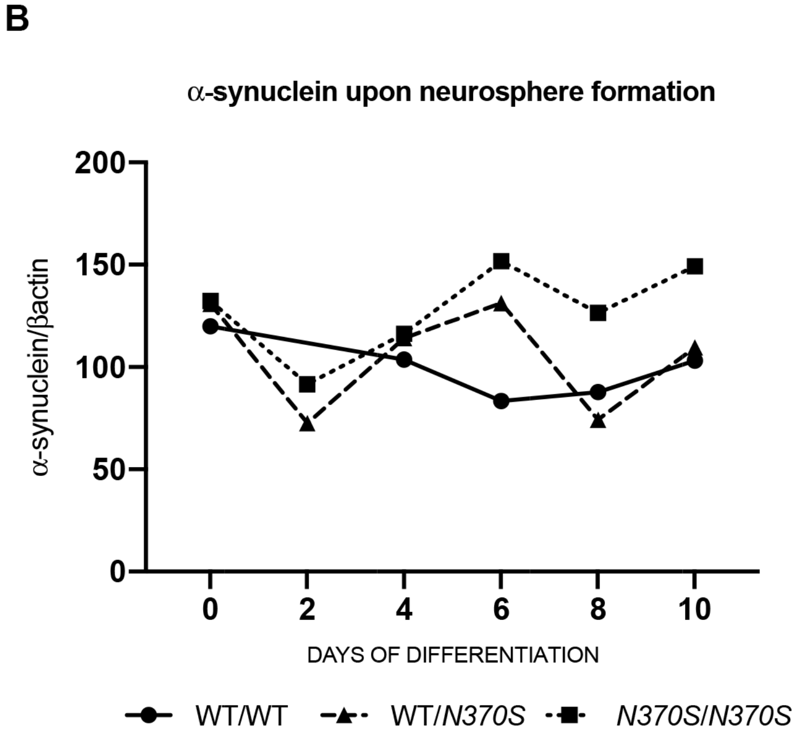

Supplementary Figure 3. Synuclein protein levels during neurospheres development. Representative blots of synuclein protein expression levels during neurospheres development (**A**) and quantification of the protein expression levels (**B**). There is no difference in a-synuclein levels at 4 days, the time point where most experiments were conducted.

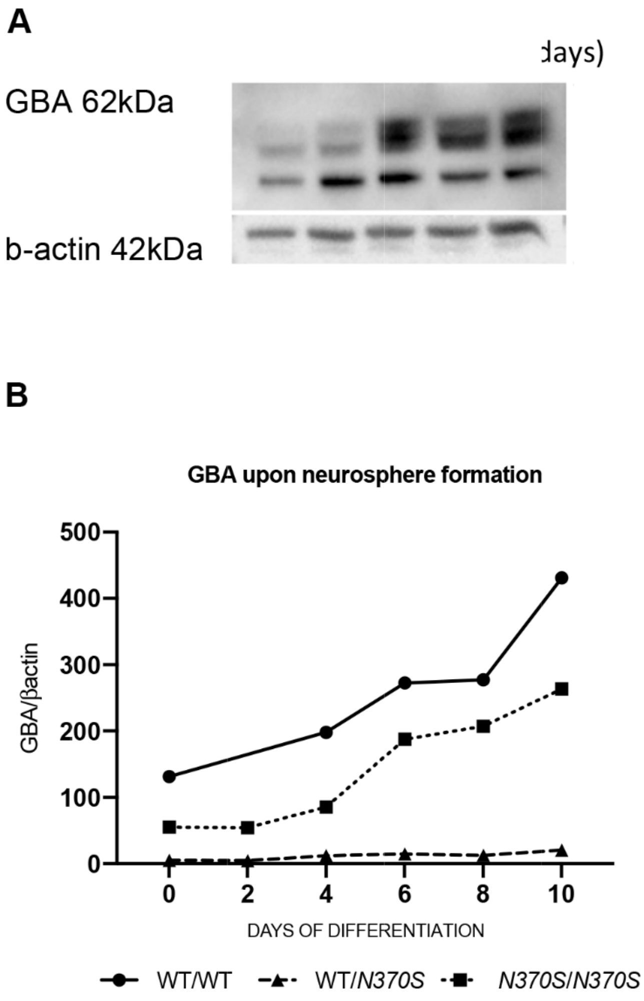

Supplementary Figure 4. (**A**). Representative blots and quantification of GBA protein levels in the control, homozygous and heterozygous lines. (**B**). Quantification of GBA protein levels over time in all the three genotypes.
