# Supplementary material for: GBA mutation promotes early mitochondrial dysfunction in 3D neurosphere models
Source: Aging (Albany NY). 2019 Nov 21;11(22):10338–55. doi: 10.18632/aging.102460 (PMC6914435; doi:10.18632/aging.102460)
Supplement: Supplementary Tables [file aging-11-102460-s001..pdf]

SUPPLEMENTARY TABLES

Supplementary Table 1. Epidemiological data of the studied cohorts at the time of biopsy.

GROUP	N	GENDER		AGE		
		MALE	FEMALE	RANGE	MEAN	SEM
wt/wt	2	1	1	56-73	64.5	±1.73
wt/Δ	2	1	1	45-75	60	±2.3
(Δ/Δ)	2	1	1	58-76	67	±1.78

No significant differences were found in age or gender between groups. wt/wt: controls, wt/*N370S* heterozygous subjects for *GBA* mutation and *N370S*/*N370S*: homozygous patients with *GBA* mutation. N: Number of cases enrolled. SEM: Standard error of the mean.

Supplementary Table 2. Raw data of all the parameters measured.

Parameter (units)				Raw data: wt/wt (n=2) MEAN	wt/wt (n=2) SEM (±)	*wt/N370S* (n=2) MEAN	*wt/N370S* (n=2) SEM (±)	*wt/N370S* (n=2) %	*N370S/N370S* (n=2) MEAN	*N370S/N370S* (n=2) SEM (±)	*N370S/N370S* (n=2) %	ANOVA
cell model characterization	neuronal markers (total n=6)	bIII-tubulin (AU)	O DOD	51,55	13,43	139,84	7,11	171,27	76,58	2,32	48,55	**0,013**
			2 DOD	95,32	71,73	315,15	71,54	230,62	174,05	83,21	82,60	0,262
			4 DOD	65,88	59,25	200,98	21,98	205,07	150,79	93,86	128,89	0,440
			6 DOD	75,73	66,91	236,28	55,15	212,00	145,21	128,56	91,74	0,524
		MAP2 (AU)	O DOD	373,98	229,95	122,12	4,40	- 67,35	210,60	56,24	- 43,69	0,502
			2 D OD	245,73	58,84	63,04	35,14	- 74,35	79,17	21,29	- 67,78	0,190
			4 DOD	49,42	9,56	0,63	0,35	- 98,73	0,79	0,21	- 98,40	0,716
			6 DOD	67,18	15,41	96,66	60,73	43,89	83,10	25,19	23,70	0,872
		Nestin (AU)	O DOD	0,14	0,07	0,03	0,03	- 76,36	0,11	0,03	- 22,00	0,380
			2 D OD	1,00	0,42	0,97	0,19	- 2,99	1,06	0,52	5,21	0,380
			4 DOD	4,37	0,35	1,81	0,38	- 58,60	1,10	0,52	- 74,70	0,989
			6 DOD	8,57	2,74	2,84	0,37	- 66,84	2,00	1,04	- 76,61	0,128
	Lysosomal enzyme activities (Total n=6)	Gcase pH 5.4/NaT (nmols/minmgprot)	O DOD	384,00	4,00	167,00	22,00	- 56,51	0,50	1,50	- 99,87	**0,001**
			2 DOD	502,50	25,50	149,50	42,50	- 70,25	14,50	1,50	- 97,11	**0,003**
			4 DOD	355,00	83,00	239,00	65,00	- 32,68	- 6,50	0,50	- 101,83	0,053
			6 DOD	651,50	65,50	438,00	140,00	- 32,77	10,50	26,50	- 98,39	**0,033**
		Gcase pH 4.5 (nmols/minmgprot)	O DOD	46,50	21,50	11,00	5,00	- 76,34	8,50	0,50	- 81,72	0,207
			2 DOD	111,50	41,50	87,00	27,00	- 21,97	54,50	6,50	- 51,12	0,469
			4 DOD	145,00	52,00	86,50	9,50	- 40,34	6,50	1,50	- 95,52	0,469
			6 DOD	195,50	1,50	120,50	54,50	- 38,36	56,00	46,00	- 71,36	0,201
		HEX	O DOD	66,50	23,50	71,50	15,50	7,52	56,50	3,50	- 15,04	0,816
			2 DOD	150,50	12,50	121,50	77,50	- 19,27	109,00	17,00	- 27,57	0,821
		ot)	4 DOD	133,50	29,50	150,00	19,00	12,36	122,00	28,00	- 8,61	0,764
			6 DOD	234,00	31,00	177,00	2,75	- 24,36	209,50	99,50	- 10,47	0,810
		b-gal	O DOD	292,00	73,00	340,50	20,50	16,61	192,50	19,50	- 34,08	0,207
			2 D OD	842,00	277,00	494,00	230,00	- 41,33	775,00	232,00	- 7,96	0,622
		ot)	4 DOD	602,00	90,00	544,00	272,50	- 9,63	528,00	133,00	- 12,29	0,956
			6 DOD	967,50	237,50	692,00	188,00	- 28,48	587,50	240,50	- 39,28	0,536
mitochondria	Mitochondrial content (Total n=9)	VDAC-1/b-actin (AU)	UT	258,64	29,63	73,96	18,69	- 71,41	338,34	88,81	30,82	0,088
			CCCP	144,48	53,99	138,43	21,85	- 4,19	251,18	58,51	73,85	0,311
		SDHA/b-actin (AU)	UT	328,90	14,77	263,85	19,86	- 19,78	831,23	234,23	152,73	0,106
			CCCP	158,72	9,02	204,41	14,98	28,79	318,22	44,52	100,49	0,055
		TFAM/b-actin (AU)	UT	116,13	1,94	120,46	0,73	3,73	240,93	172,55	107,47	0,647
			CCCP	56,12	10,94	224,77	6,17	300,52	141,74	6,76	152,57	**0,002**
	ATP Levels (Total n=9)	ATP PmolATP/ul*mgprot)	0 HRS	0,01	0,01	0,01	0,00	- 29,34	0,01	0,01	- 21,32	0,052
			4 HRS	0,01	0,01	0,01	0,00	- 24,91	0,01	0,00	- 28,16	**0,005**
			16 HRS	0,00	0,00	0,00	0,00	66,68	0,01	0,00	190,40	**0,001**
			24 HRS	0,00	0,00	0,00	0,00	59,50	0,01	0,01	253,52	**0,005**
Autophagy	Regulation (Total n=6)	TFEB/GDAPH (AU)	UT	0,01	0,00	0,02	0,00	88,77	0,03	-	135,47	0,084
			CCCP	0,01	0,00	0,01	0,00	31,73	0,02	-	53,94	**0,030**
		PGC1D/GDAPH (AU)	UT	0,08	0,00	0,12	0,00	49,71	0,11	-	40,87	**0,014**
			CCCP	0,57	0,11	2,76	0,17	382,94	0,44	-	- 23,21	**0,015**
	Autophagic flux (Total n=9)	P62/bACTIN (AU)	UT	252,42	30,69	137,08	16,69	- 45,69	193,86	56,29	- 23,20	0,184
			BAF	924,84	555,57	602,24	149,36	- 34,88	887,29	145,28	- 4,06	0,776
		LC3B2/bACTIN (AU)	UT	1.499,54	754,45	559,01	253,21	- 62,72	674,63	176,19	- 55,01	0,316
			BAF	4.385,22	1.404,53	2.624,67	949,57	- 40,15	3.193,41	364,23	- 27,18	0,446

wt/wt: controls, wt/*N370S* heterozygous subjects for *GBA* mutation and *N370S*/*N370S*: homozygous patients with *GBA* mutation. DOD: days of differentiation, UT: untreated, BAF: bafilomycin.
